# Adjuvant gemcitabine after resection of pancreatic cancer without significant difference in overall survival: a retrospective cohort study

**DOI:** 10.1097/MS9.0000000000000854

**Published:** 2023-05-20

**Authors:** Katrin Bauer, Peter Büchler, Doris Henne-Bruns, Giulia Manzini

**Affiliations:** a Department for General and Visceral Surgery, Clinic of Kempten, Kempten; b Department of General and Visceral Surgery, University Hospital of Ulm, Ulm, Germany; c Department of Visceral Surgery, Cantonal Hospital of Aarau, Aarau, Switzerland

**Keywords:** adjuvant chemotherapy, ductal adenocarcinoma, gemcitabine, guidelines, pancreatic carcinoma

## Abstract

International guidelines recommend adjuvant chemotherapy after resection of pancreatic adenocarcinoma. The administration of gemcitabine has become part of the interdisciplinary treatment concept. The authors aim to prove whether the benefit in overall survival (OS) reported in randomized controlled trials (RCTs) could be reached also for patients treated in their department.

**Materials and methods:**

The authors retrospectively analyzed the OS of all patients who underwent pancreatic resection at their clinic because of ductal adenocarcinoma between January 2013 and December 2020 in dependence on adjuvant treatment with gemcitabine.

**Results:**

Overall 133 pancreatic resections were performed between 2013 and 2020 due to malignant pancreatic pathology. Seventy-four patients had ductal adenocarcinoma. Forty patients received adjuvant gemcitabine chemotherapy postoperatively, 18 patients underwent only surgical resection, and 16 patients received other chemotherapy regimens. The authors compared the group receiving adjuvant gemcitabine (*n*=40) with the group undergoing surgery alone (*n*=18). The median age was 74 years (range: 45–85), and the median OS was 16.5 months [95% confidence interval (CI) 13–27]. Follow-up time was at least 23 months (range 23–99). No statistically significant difference in median OS was observed in the group who received adjuvant chemotherapy compared to the operation-only group [17.5 months (range: 5–99, 95% CI 14–27) versus 12.5 months (range: 1–94, 95% CI 5–66), *P*=0.75].

**Conclusion:**

OS with and without adjuvant chemotherapy with gemcitabine was comparable to the results of those RCTs which serve as the basis of guideline recommendations. However, the analyzed patient cohort did not profit significantly from the adjuvant treatment.

## Graphical abstract

The graphical abstract is shown in the figure.

**Figure:**
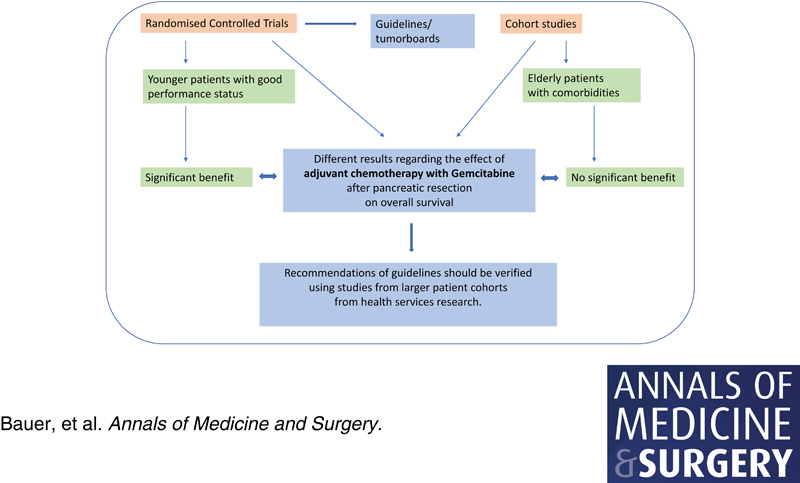

## Introduction

Highlights

- Guidelines recommend adjuvant chemotherapy after pancreatic cancer resection.
- Gemcitabine-mono is recommended for patients with lower performance status.
- The analyzed patient cohort did not benefit significantly from gemcitabine.
- Adverse side effects were frequent and often led to the discontinuation of treatment.
- Recommendations of guidelines cannot be transferred to our patient cohort.

The incidence of pancreatic carcinoma is increasing worldwide. It is supposed that this type of carcinoma will represent the second most frequent cause of death in the U.S. in 2030^1^. For Germany, the Institute of Robert Koch reports an incidence of about 20 000 pancreatic cancer cases in 2019, with an estimation of 22 000 cases in 2023 (https://www.rki.de/DE/Content/Gesundheitsmonitoring/Krebsregisterdaten/krebs_node.html). The median age at diagnosis is 76 years for women and 72 years for men. The prognosis is still poor, although many efforts have been made to improve the overall survival (OS) of patients with adenocarcinoma of the pancreas^2^. Surgical resection is still the only curative therapeutic option in case of pancreatic carcinoma. Based on several randomized controlled studies^3,4^, the international guidelines actually recommend adjuvant chemotherapy after resection of pancreatic cancer, regardless of the tumor and the lymph-node status (http://www.nccn.org/professionals/physician_gls/pdf/pancreatic.pdf)^5,6^. Depending on the general condition and comorbidities of the patient, several chemotherapy protocols are recommended^4,7,8^.

Pancreatic carcinoma is quite resistant toward chemotherapy compared with other tumor diseases^9^. Therefore, a prolongation of median survival of only a few months can be reached. Until now, immunotherapy, which has developed to a new standard for many tumor entities in the last years, has been less successful in the case of pancreatic carcinoma. Target-oriented individualized therapy based on molecular aberrations proved to be difficult, because the dominant driver mutation KRAS, which exists in 90–95% of the patients, cannot be treated target-oriented successfully yet.

The S3-German guideline^10^ recommends adjuvant administration of gemcitabine after R0-resection in the UICC stages I–III with a strength of grade ‘A’ according to the Oxford grading system. Chemotherapy should begin within 6 weeks after surgery and should be performed for 6 months. Gemcitabine is also recommended in cases of R1-resections (grade B). The administration of FOLFIRINOX is recommended in the metastatic situation for patients with a good performance status or in a neoadjuvant concept for patients with primarily unresectable tumors included in clinical trials. These recommendations correspond with the ESMO-guideline^5,^ the NCCN-guideline (http://www.nccn.org/professionals/physician_gls/pdf/pancreatic.pdf), SEOM-guideline^6,^ etc.

As known, the patients included in randomized controlled trials (RCTs), on which meta-analyses and guidelines rely, are highly selected with stringent inclusion and exclusion criteria and do not match the ‘mean patient’ that caregivers have to treat routinely^11^. For example, data about the effects of drugs in selected patients groups, like elderly patients, are lacking^12^. Moreover, new drugs are administered for a limited period of time in RCTs and data about risk and benefit of longer use are not present.

In this regard, the aim of our study was to analyze the patients’ outcomes in our clinic regarding the question of whether adjuvant treatment with gemcitabine as a first-line mono-therapy after resection of ductal adenocarcinoma of the pancreas could improve OS.

## Materials and methods

By using the hospital information system, the data of all patients who underwent a curative intended pancreatic resection for ductal adenocarcinoma between January 2013 and December 2020 were collected retrospectively in order to analyze OS in dependence on adjuvant treatment with gemcitabine. As a public, tertiary-care hospital, our hospital is certified by the German Cancer Society for the treatment of pancreatic carcinoma since 2013. Clinical examination, computed tomography (CT) scan of the abdomen and thorax, and the value of CA (carbohydrate antigen) 19-9 are the basic diagnostics. Some patients received a histological confirmation before surgery via endoscopic retrograde cholangiopancreatography (ERCP). All patients were presented in the interdisciplinary tumor board before and after resection. Surgery was performed by two hepatobiliary/pancreatic surgeons, with an average number of pancreatic interventions of about 25 per year. Pylorus-preserving partial pancreatoduodenectomy with lymphadenectomy or pancreatic tail resection with splenectomy and lymphadenectomy were performed. In some cases, extended resection of other organs like the stomach, colon, portal vein, etc. was necessary. Histological examination was done according to the American Joint Committee on Cancer (AJCC)^13^. The decisions of the interdisciplinary tumor board were made on the basis of German guidelines for pancreatic cancer^10^. These guidelines recommend adjuvant chemotherapy with gemcitabine for pancreatic cancer in the UICC stages I–III. Six cycles of gemcitabine should be administered every 4 weeks, consisting of three weekly infusions of gemcitabine (1000 mg/m^2^) followed by a one-week break. Since 2016, fitter patients received a combination therapy with gemcitabine and capecitabine while the young and very fit patients received FOLFIRINOX. The applied algorithm divided the patients into four groups:

Very frail: no adjuvant therapy

Slow go: Gemcitabine mono

Relatively fit without severe comorbidity: Ggemcitabine + Capecitabine

Really fit: FOLFIRINOX

Chemotherapy was administered either in the oncology department of our hospital or on an outpatient basis by oncologists. In cases of relapse or metastasis, the patients partly received second-line or even third-line chemotherapies with different regimens.

The preoperative, intraoperative, and postoperative characteristics of the patients were evaluated. All patients received intraabdominal drainage at the end of the surgical intervention.

In order to detect the pancreatic fistulas, the lipase levels of the drainage fluid were evaluated only in case of suspicious secretion. In cases of fever or signs of inflammation, a CT scan was performed and, when necessary, a drain was interventionally placed in detected fluid collections. This corresponds to a surgical complication grade 3a according to the Clavien–Dindo classification^14^.

The manuscript was edited according the 17-item checklist of the STROCSS Guideline published by Mathew and Agha^15^ in 2017.

The study was registered in www.researchregistry.com with the registration unique identifying number (UIN) 8507 and the following hyperlink: https://www.researchregistry.com/browse-the-registry#home/registrationdetails/637f8b34a2e1a90021459b99/.

## Statistical analysis

Values are presented as the mean (±standard deviation) and median (range) for continuous variables. Median survival is reported with the 95% confidence interval (CI). Dichotomic variables are presented as absolute number as well as percent. A two-sided *P* value of less than 0.05 was considered statistically significant. Survival curves were obtained with the Kaplan–Meier method according to chemotherapy (yes or no). Missing values were less than 5% in the data set, and no imputation strategies were used. All calculations were conducted using R Project for Statistical Computing (The R Foundation, Version 3.1.0, Vienna, Austria), in particular the libraries ‘survival’ and ‘splines’ were used. Survival analysis was performed with the function ‘survfit’ and survival curves were compared with the function ‘survdiff’. Survival analysis was not adjusted for other variables because of the small sample size in order to avoid overfitting.

All patients were followed up for at least 23 months (range: 23–99).

## Results and discussion

**Figure 1:**
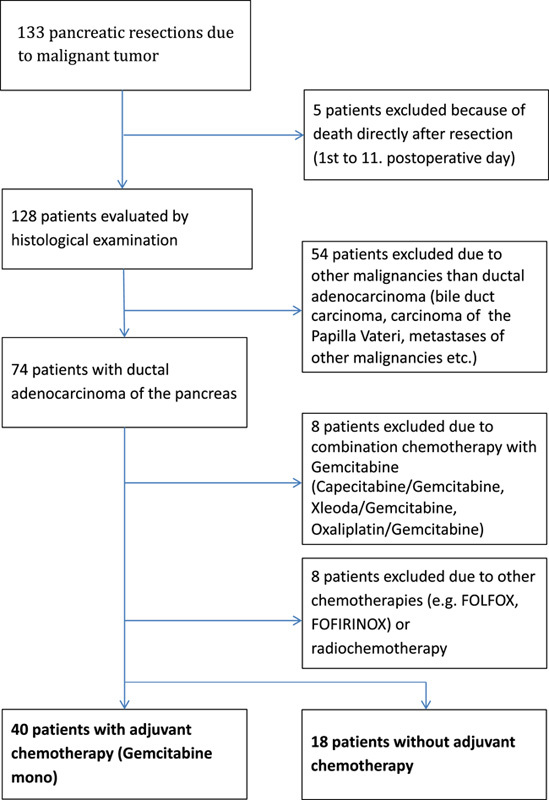
Patients’ flowchart.

A total of 133 patients underwent a pancreatic resection because of malignancy between January 2013 and December 2020. Five patients died immediately after surgery (range first day until 11th day). The histological findings of 128 patients were evaluated. Patients with other malignancies than ductal adenocarcinoma of the pancreas (54 patients) or patients with other adjuvant treatments than gemcitabine (16 patients) were excluded in order to have a unique treatment group (Fig. 1).

The interdisciplinary tumor board recommended an adjuvant gemcitabine-based chemotherapy to all of the 58 patients included in the study. Eighteen patients (31%) did not receive any chemotherapy and 40 patients (69%) received adjuvant chemotherapy with gemcitabine. Low performance status due to age or comorbidities and/or persisting pancreatic fistula were the most frequent reasons not to administer adjuvant chemotherapy. Patient’s wish and progressive disease were less common.

In order to find a survival benefit of the gemcitabine treatment, we compared the 40 patients who received gemcitabine postoperatively as monotherapy with the 18 patients who underwent only surgical resection. Preoperative and postoperative patients’ characteristics are shown in Table 1. In addition, the comorbidities were summarized in Table 2: hypertension, cardiac, and metabolic diseases were the most common.

**Table 1:** Patient characteristics

	All patients	Gemcitabine group	Operation-only group
Number of patients	58 (100%)	40 (69%)	18 (31%)
Age at diagnosis			
Median (range)	74 years (45–86)	71 (45–85)	76 (61–85)
Gender			
Female	31 (53.5%)	22 (55%)	9 (50%)
Male	27 (46.5%)	18 (44%)	9 (50%)
ASA Classification			
1	4 (6.9%)	4 (10%)	0 (0%)
2	33 (56.9%)	19 (47.5%)	14 (77.8%)
3	21 (36.2%)	17 (42.5%)	4 (22.2%)
Tumor location			
Head	45 (77.6%)	35 (87.5%)	10 (55.6%)
Corpus	2 (3.5%)	1 (2.5%)	1 (5.6%)
Tail	11 (19. %)	4 (10%)	7 (38.9%)
Procedure			
Pylorus-preserving Whipple	46 (79.3%)	36 (90%)	10 (55.6%)
Tail resection with splenectomy	12 (20.7%)	4 (10%)	8 (44.4%)
UICC Stage			
IA	2 (3.5%)	2 (5%)	
IB	11 (19.0%)	7 (17.5%)	4 (22.2%)
IIA	1 (1.7%)		1 (5.6%)
IIB	31 (53.5%)	24 (60%)	7 (38.9%)
III	7 (12.1%)	4 (10%)	3 (16.7%)
IV	6 (10.3%)	3 (7.5%)	3 (16.7%)
Fistula rate	12 (20.7%)	6 (15%)	6 (39%)

ASA, American Society of Anesthesiologists; UICC, Union Internationale Contre le Cancer.

**Table 2:** Comborbidities

	All patients (*n*=58)	Gemcitabine (*n*=40)	Surgery only (*n*=18)
Cardial (aortic valve stenosis, coronary heart disease, atrial fibrillation, heart failure, dilatative cardiomyopathia)	17 (29.3%)	14 (35%)	3 (16.7%)
Pulmonal (chronic bronchitis, COPD, nicotine abuse, asthma bronchiale)	9 (15.5%)	3 (7.5%)	6 (33.3%)
Metabolic diseases (diabetes mellitus, porphyria, Adipositas)	21 (36.2%)	15 (37.5%)	6 (33.3%)
Vascular (aortic aneurysm, carotid stenosis, peripheral arterial occlusive disease)	4 (6.9%)	4 (10%)	0
Hypertension	25 (43.1%)	17 (42.5%)	8 (44.4%)
Renal (renal insufficiency)	2 (3.5%)		2 (11.1%)
Psychological (depression, anxiety disorder)	5 (8.6%)	3 (7.5%)	2 (11.1%)
Neurological (polyneuropathy, dementia, stroke)	4 (6.9%)	1 (2.5%)	3 (16.6%)
Autoimmune diseases (rheumatoid arthritis, fibromyalgia, Morbus Bechterew, Myasthenia gravis)	4 (6.9%)	4 (10%)	
Hematological (anemia, factor 5 deficiency	3 (5.2%)	3 (7.5%)	

79% of all tumors were located in the pancreatic head. Therefore, pylorus-preserving Whipple’s procedure was the most performed surgical intervention. All pancreatic resections were performed by two surgeons. The most common tumor stage according to the UICC was IIB. More than 70% of the patients received adjuvant gemcitabine. The time of follow-up was at least 23 months (range 23–99), so 41 events were recorded (seven patients alive in 5/2021). The median age of the patients at the time of diagnosis was 74 years (71 in case of chemotherapy and 76 in case of no chemotherapy). The group of patients who received gemcitabine was in median 5 years younger than the patients who only received surgery.

**Figure 2:**
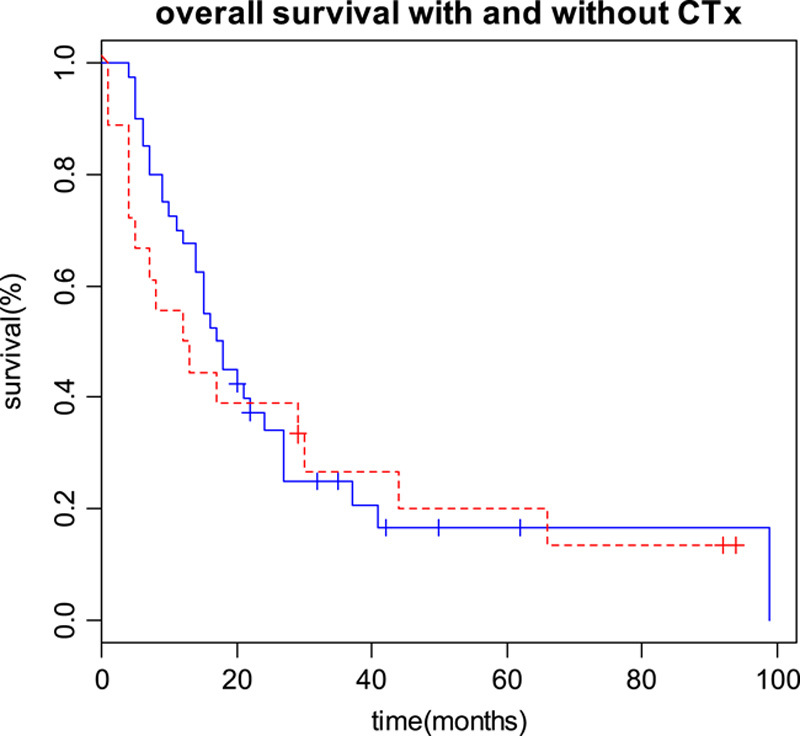
Kaplan–Maier curve for the two treatment groups, red=without adjuvant chemotherapy, blue=with adjuvant chemotherapy.

Median OS time of all patients was 16.5 months (95% CI 13–27 months). Median survival in the chemotherapy group was 17.5 months (95% CI 14–27 months) and in the surgery-only group 12.5 months (95% CI 5–66 months). Survival curves are shown in Figure 2. No significant difference in survival was observed between the treatment groups (*P*=0.75).

Pancreatic fistula was the most common postoperative complication, followed by pulmonal complications (see Table 3). Operative reintervention due to pancreatic fistula was not necessary. All fistulas solved in variable time, and the drainage could be removed within the hospital stay or the following weeks. No drainage-associated bleeding was observed. In cases of fever or inflammation, a CT scan was performed, and a drain was placed in a potential collection.

**Table 3:** Intraoperative and postoperative complications

	All patients (*n*=58)	Gemcitabine (*n*=40)	Surgery only (*n*=18)
Intraoperative			
** **Bleeding/transfusion	1 (1.7%)	1 (2.5%)	
** **Cutting renal vessels	1 (1.7%)	1 (2.5%)	
Postoperative			
Cardial (decompensated cardiac insufficiency, angina pectoris)	3 (5.2%)	2 (5%)	1 (5.6%)
Pulmonal (pulmonary embolism, pneumonia, ARDS, pneumothorax)	6 (10.4%)		6 (33.3%)
Wound healing	4 (6.9%)	3 (7.5%)	1 (5.6%)
Pancreatic fistula	12 (20.7%)	6 (15%)	6 (33.3%)
Neurological (stroke, delir, nerve lesion)	3 (5.2%)		3 (16.7%)
Arosion bleeding	1 (1.7%)	1 (2.5%)	
Gastrointestinal (ileus, paralysis, stenosis of the gastroenteral anastomosis, Clostridia infection)	5 (8.6%)	3 (7.5%)	2 (11.1%)
Renal (acute kidney failure)	1 (1.7%)	1 (2.5%)	
Septic shock	2 (3.4%)	2 (5%)	
Lymphatic fistula	3 (5.2%)	3 (7.5%)	
Urinary tract infection	2 (3.4%)	1 (2.5%)	1 (5.6%)
Vascular (closure of the cubital artery)	1 (1.7%)	1 (2.5%)	

However, quite a different rate of pancreatic fistula was seen in the two treatment groups: 15% in the group with adjuvant chemotherapy and 39% in the surgery-only group (Table 1).

Twenty-three of 40 patients (57.5%) did not receive all six recommended cycles due to adverse events. Gastrointestinal symptoms, deterioration of general condition, and progression were the most common reasons for discontinuation of therapy. Tables 4 and 5 provide an overview of the side effects and the distribution of the cycles received.

**Table 4:** Side effects of gemcitabine

	Number of patients (*n*=23)	Proportion in %
Abdominal pain, nausea, vomiting, subileus, constipation	7	30.4
Gemcitabine-associated pneumonitis, respiratory insufficiency	2	8.7
Mouth sore	1	4.4
Cardiac decompensation	1	4.4
Weight loss, weakness, loss of appetite, asthenia, chills, psychological problems	6	26.1
Polyneuropathy	1	4.4
Hematopoietic insufficiency (leukopenia, thrombocytopenia)	3	13.0
Skin rash	1	4.4
Stomatitis, Chelitis	1	4.4
Progress (hepatic filiae, peritoneal carcinomatosis, ascites, cholestasis)	8	34.8

**Table 5:** Distribution of the administered cycles of gemcitabine

Number of CTX cycles		1	2	3	4	5	6
Number of patients (%)		4 (10%)	8 (20%)	6 (15%)	3 (7.5%)	2 (5%)	17 (42.5%)

CTX, Chemotherapy.

Guidelines are based on meta-analyses, RCTs, and cohort studies. It is of crucial importance to evaluate guidelines with respect to the possibility of applying their recommendations to daily praxis. Patients recruited for randomized studies often are younger and show a better performance status than patients of everyday clinic^11,12,16^.

German guidelines regarding exocrine pancreatic carcinoma^10^ were established in 2013 and revised in 2018. The recommendation of adjuvant administration of gemcitabine is based on a few small current RCTs^4,7,17,18^. In our clinic, all patients with pancreatic carcinoma are discussed in the interdisciplinary tumor board whose decision is based on German guidelines. We aimed to investigate if the results of the studies on which guidelines’ recommendations rely are applicable to the situation in a tertiary-care hospital.

While most studies report a 5-year OS rate of only 20% for patients with resectable ductal adenocarcinoma of the pancreas^3,4^, patients with carcinomas of the papilla vateri show a 5-year survival rate of 30–40%^19,^ because these tumors are often diagnosed earlier due to the obstruction of the bile duct. In order to have comparable groups, only patients with ductal adenocarcinoma were included in this study.

In order to compare the results from our patient cohort regarding age, OS, adverse effects of gemcitabine, fistula rate, etc., with the results of the few RCTs which analyzed adjuvant treatment with gemcitabine after pancreatic resection, the best-known of these studies will be described below:

The ESPAC-1 study^3^ investigated whether an adjuvant treatment (chemotherapy for 6 months with 5-FU and D-L folinic acid or radiotherapy with 40 Gray and 5-FU as radiosensitizer or a combination therapy of both) could improve the 2-year survival of patients with resected pancreatic cancer. Two-hundred and fifty-six patients were included into four treatment groups (observation, radiotherapy only, chemotherapy only, and radio+chemotherapy). The median age of all patients was 61 years. Neoptolomos *et al*.^3^ concluded that 5-FU-based chemotherapy can significantly increase the OS of patients with resectable pancreatic cancer. The median survival time was 20.1 months among the patients who received chemotherapy (chemotherapy alone or radiotherapy + chemotherapy) and 15.5 months among the patients without chemotherapy (observation or radiotherapy). The design of the study was a confusing two-factorial design, so that the particular treatment groups could not be compared with each other^20^. Of the 122 patients randomly assigned to receive chemotherapy, 21 (17%) did not receive any chemotherapy; 24% of the patients had postoperative complications. With this study, adjuvant chemotherapy after pancreatic resection was established.

Also, the ESPAC-3 study (version 2)^17^ analyzed the efficacy of adjuvant chemotherapy after resection of pancreatic cancer. The administration of 5-FU plus folinic acid was compared with gemcitabine in 1088 patients all over the world. While treatment with 5-FU plus folinic acid was as effective as administration of gemcitabine, adverse effects were significantly lower under treatment with gemcitabine. The median age at diagnosis was 63 years. Further, it was shown that start of therapy could take place up to 12 weeks after resection. The determining prognostic factor was not the early start but the complete administration of all six cycles of adjuvant therapy. Median OS was 23.0 months for 5-FU and 23.6 months for gemcitabine treatment.

The multicenter, open-label CONKO-001 study^4^ investigated whether adjuvant chemotherapy with gemcitabine could influence the survival of patients with pancreatic cancer after resection. An improved 5-year survival rate of 11% after administration of adjuvant gemcitabine was reported. The median age of the patients was 62 years. Long-term results showed a median OS time of 22.8 months in the chemotherapy group and 20.8 months in the surgery-only group. Allover survival after 5 years was 20.7% with adjuvant therapy versus 10.4% without adjuvant therapy (*P*=0.01). This result was significant due to the large number of patients enrolled and the long follow-up time. Among the 186 patients recruited into the gemcitabine-therapy group, only six patients could not turn up for chemotherapy within the 6-week interval due to postoperative complications (complication rate: 3.2%).

A Japanese working group examined the effect of adjuvant chemotherapy with gemcitabine in a study with a quite similar design to that of the CONKO-001 study. In 2009, the study by Ueno *et al*.^18^ recruited 118 patients and demonstrated a significant advantage in disease-free survival in the chemotherapy group. The results for the OS were very similar to the CONKO study: median OS was 22.3 months in the gemcitabine group versus 18.4 in the surgery-only group, but there was a lack of significance presumably due to the smaller group of patients enrolled.

Since 2016, patients with a better performance status received a combination therapy of capecitabine and gemcitabine on the basis of the ESPAC-4 study^7^. Between 2008 and 2014, 732 patients were randomized. The median age was 65 years. The combination therapy led to a benefit of median OS (28.0 months vs. 25.5 months, *P*=0.032) and 5-year survival (28.8 vs. 16.3%) while toxicity was tolerable. The advantages were seen more clearly in the R0-group than in the R1-group. So the combination therapy became the new standard for adjuvant chemotherapy after resection of pancreatic carcinoma for fitter patients without severe comorbidity.

Only few patients qualify for an even more aggressive chemotherapy regime. In the French–Canadian PRODIGE-24/CCTG-PA.6-Study, palliative patients with good performance status were treated with a combination therapy of oxaliplatin, irinotecan, and 5-FU (FOLFIRINOX) which showed a superior outcome compared with gemcitabine^21^. In the adjuvant situation, a modified regime with less irinotecan and without a 5-FU-Bolus was administered (mFOLFIRINOX^8^). The 493 randomized patients of the French–Canadian PRODIGE-24/CCTG-PA.6-Study received either mFOLFIRINOX or gemcitabine. The median age at diagnosis was 63 years. Inclusion criteria were very good performance status without comorbidity and a CA 19-9 value under 180 U/l. Administration of mFOLFIRINOX led to a significant improvement in disease-free survival of 21.6 months (gemcitabine: 12.8 months) and a median OS of 54.4 months (gemcitabine: 35.0 months). The toxicity of mFOLFIRINOX was higher but tolerable.

In the JASPAC 01 Study^22,^ gemcitabine was compared with oral fluoropyrimidin derivate S-1 in 385 Japanese patients with R0-resection. Five-year survival rate was 44.1% in the patients treated with S-1 compared with 24.4% in patients treated with gemcitabine.

Comparing our results with all these studies, it is obvious that patients treated in our hospital were on average more than 10 years older than those treated within the mentioned trials (e.g. CONKO-001 study, median age: 62 years^4^). This difference in the patients’ age between reported studies and our own retrospective analyses, therefore, explains a higher probability of preexisting comorbidities and therefore a higher postoperative overall complication rate.

While gemcitabine is considered well tolerated, 23 of 40 patients (57.5%) did not receive all six recommended cycles due to adverse events. In the CONKO study^4,^ 38% of the patients allocated to treatment with gemcitabine had to discontinue the therapy, while Ueno *et al*.^18^ report a discontinuation of gemcitabine treatment of only 24%.

Pancreatic fistula was more often observed in the group without chemotherapy although none of the patients had to be reoperated due to pancreatic fistula. There is an obvious trend that pancreatic fistula can not only delay but prohibit adjuvant therapy because chronic abdominal infection due to the fistula affects the performance status and the convalescence of the patient. However, the subgroups are too small to evaluate significant results.

In spite of partly extended resection and administration of adjuvant chemotherapy, survival rates in the evaluated collective remain poor. These findings are comparable with the results of the ESPAC-1^3,^ the CONKO-001^4,^ and the ESPAC-3^17^ study.

The median observed prolongation of OS was 5 months in the patients who received gemcitabine and was not significant. In large patient collectives and long follow-up periods, results can become significant as could be seen from the results of the CONKO-001 study^4,^ where a survival difference of 2 months between the treatment groups finally reached significance after a follow-up period of 8 years.

We already analyzed the quality of the ESCPAC-1 study^20^ and the CONKO-001^23^ study to evaluate their validity and risk of bias according to the Cochrane Risk of Bias Tool and came to the conclusion that their validity is not sufficient enough to serve as the basis of guidelines recommendations.

In diverse meta-analyses^24–27^ that compared postoperative gemcitabine therapy with other therapy combinations, all authors concluded that the effects of chemotherapies are altogether poor.

The limitations of this study are the retrospective design and the small number of patients with ductal adenocarcinoma, which do not allow sufficient power. The low power should be taken into account by the interpretation of the *P* value for the comparison of the median OS in the operation-only group versus the group receiving chemotherapy (according to sample size calculation, 678 patients would be needed). Whenever possible, RCTs are the best way to achieve statistically significant results with enough power.

The decision whether to administer gemcitabine or not was not made by randomization but in discussion between the oncologist and patient regarding the expected benefit and the risks of side effects after the recommendation of the tumor board had been given. Certainly, there is a bias to a certain extent that the tumor board’s recommendation regarding adjuvant therapy is more restrictive in an elderly and comorbid patient.

Another limitation is that the grade of severity of pancreatic fistula was not scored by a grading into A/B/C postoperative pancreatic fistula (POPF) according to the International Study Group on Pancreatic Surgery (ISGPS) definition of 2005^28^. The value of lipase was not determined routinely but only in case of tryptic secretion or in case of draining a collection via CT scan postoperatively. So particularly, grade A POPF was probably underestimated in both groups. As there was no reoperation due to pancreatic fistula, the majority of reported fistulas were grade B.

However, surgery was performed by only two surgeons with standardized methods. Since 2013, our clinic is certified by the German Cancer Society for the treatment of carcinomas of gastric, colorectal, and pancreatic carcinomas. Therefore, the in-house patient treatment is more standardized than in the RCTs, where the participating clinics enrolled about one patient per year on average (CONKO-001: 0,7 patients^4,^ ESPAC-1: 1,5 patients^3,^ Ueno *et al*.: 0,3 patients^18^).

## Conclusion

Administration of gemcitabine as adjuvant chemotherapy after resection of ductal pancreatic adenocarcinoma is recommended in the German guideline for patients with lower performance status. Our study failed to demonstrate that adjuvant therapy with gemcitabine improves OS in our patient cohort. Adverse side effects were frequent and often led to discontinuation of the treatment.

To decide whether to administer adjuvant gemcitabine, the results of this study should be verified using studies from larger patient cohorts from health services research.

## Ethical approval

This research study was conducted retrospectively from data obtained for clinical purposes. According to the consultation (via e-mail) of the State Medical Association, it was not necessary to obtain a decision from the Ethics Commission for this retrospective analysis of internal hospital data. The request to see if an ethics vote was needed was made via e-mail back in 2018 when the study was being planned. So there is no judgment’s reference number.

## Consent

According to the consultation (via e-mail) of the State Medical Association, written consent was not necessary for the retrospective analysis of internal hospital data.

## Sources of funding

This study was not funded.

## Author contribution

B.K. and M.G.: contributed equally to this work; B.K.: collected data; B.K., H.-B.D., B.P., and M.G.: designed the study; B.K., H.-B.D., and M.G.: analyzed the data; M.G.: worked out the statistical analysis and was responsible for software; B.K. and M.G.: wrote the manuscript (original draft); H.-B.D.: responsible for review and editing and performed the supervision of the project: B.K., B.P., H.-B.D., and M.G.: responsible for the interpretation of results and the critical revision of the manuscript.

## Conflicts of interest disclosure

The authors declare that they have no conflicts of interest.

## Research registration unique identifying number (UIN)

1. Registry used: Researchregistry.com.
2. Unique identifying number: 8507.
3. Hyperlink to the specific registration: https://www.researchregistry.com/browse-the-registry#home/registrationdetails/637f8b34a2e1a90021459b99/

## Guarantor

Katrin Bauer.

## Provenance and peer review

Not commissioned, externally peer-reviewed.
